# Prevalence of Down's Syndrome in England, 1998–2013: Comparison of linked surveillance data and electronic health records

**DOI:** 10.23889/ijpds.v5i1.1157

**Published:** 2019-03-19

**Authors:** JC Doidge, JK Morris, KL Harron, S Stevens, R Gilbert

**Affiliations:** 1 UCL Great Ormond Street Institute of Child Health, University College London, London, WC1N 1EH, UK; 2 Intensive Care National Audit and Research Centre, London, WC1V 6AZ, UK; 3 Population Health Research Institute, St George's University of London, London, SW17 0RE, UK; 4 Public Health England, London, SE1 6LH, UK; 5 Health Data Research UK, University College London, London, NW1 2DA, UK

## Abstract

**Introduction:**

Disease registers and electronic health records are valuable resources for disease surveillance and research but can be limited by variation in data quality over time. Quality may be limited in terms of the accuracy of clinical information, of the internal linkage that supports person-based analysis of most administrative datasets, or by errors in linkage between multiple datasets.

**Objectives:**

By linking the National Down Syndrome Cytogenetic Register (NDSCR) to Hospital Episode Statistics for England (HES), we aimed to assess the quality of each and establish a consistent approach for analysis of trends in prevalence of Down’s syndrome among live births in England.

**Methods:**

Probabilistic record linkage of NDSCR to HES for the period 1998–2013 was supported by linkage of babies to mothers within HES. Comparison of prevalence estimates in England were made using NDSCR only, HES data only, and linked data. Capture-recapture analysis and quantitative bias analysis were used to account for potential errors, including false positive diagnostic codes, unrecorded diagnoses, and linkage error.

**Results:**

Analyses of single-source data indicated increasing live birth prevalence of Down’s Syndrome, particularly in the analysis of HES. Linked data indicated a contrastingly stable prevalence of 12.3 (plausible range: 11.6–12.7) cases per 10 000 live births.

**Conclusion:**

Case ascertainment in NDSCR improved slightly over time, creating a picture of slowly increasing prevalence. The emerging epidemic suggested by HES primarily reflects improving linkage within HES (assignment of unique patient identifiers to hospital episodes). Administrative data are valuable but trends should be interpreted with caution, and with assessment of data quality over time. Data linkage with quantitative bias analysis can provide more robust estimation and, in this case, stronger evidence that prevalence is not increasing. Routine linkage of administrative and register data can enhance the value of each.

**Keywords:**

Down’s syndrome; data linkage; disease surveillance; linkage error; electronic health records; prevalence

## Key messages

- Register and administrative data both indicated an increasing prevalence of Down’s syndrome among live births in England, but linked data suggest a stable prevalence.
- Analysis of Hospital Episode Statistics for England can be severely biased by linkage errors in the assignment of patient identifiers (‘HESID’) to hospital episode records, particularly when using birth episodes prior to 2009. Many administrative datasets can be similarly affected by errors in internal linkage.
- Linkage error is difficult to measure but quantitative bias analysis can be used to reflect plausible assumptions about its potential impact on an analysis.
- Linked data can provide more robust evidence for disease surveillance than single-source registry or administrative data and can support analyses involving changes in data collection systems.
- Linkage between datasets can be enhanced by identifying familial links within datasets, such as between mothers and children in Hospital Episode Statistics.

## Introduction

Congenital anomalies are a major cause of infant mortality, childhood morbidity and long-term disability, affecting over 1 in 50 children worldwide [1]. Accurate surveillance of congenital anomalies is essential to ensure that the right services are available to treat affected children, to provide reliable information on outcomes for prospective parents faced with difficult decisions in early pregnancy, to guide prevention programmes and for research into pregnancy and birth characteristics associated with anomalies. Congenital anomaly registries have been set up to collect accurate information for the surveillance of all anomalies [2]. Longitudinal population data routinely collected for administrative purposes (e.g. payments by health insurers and universal healthcare systems such as the NHS) offers an additional resource for identification of cases, information about long-term outcomes, and data on comparator populations to support analysis of aetiology and risk. However, the quality of administrative data is variable and its suitability for research applications requires careful evaluation. Linking data from independent sources of information about the same condition can be used to assess the quality of each source and to estimate the total number of cases, including those not detected by either source. Data linkage brings additional complexities, particularly around linkage error and integration of multiple sources of potentially conflicting information. In this article, we compare several possible approaches to analysis of linked population data, which we hope will provide methodological insights for population data science beyond their present application to Down's syndrome [3].

The National Down's Syndrome Cytogenetic Register (NDSCR) began in 1989 and collected all cytogenetic or DNA reports of trisomy 21 and the cytogenetic variants occurring in England and Wales [4]. In 2015 the NDSCR was incorporated into the National Congenital Anomaly and Rare Disease Registration Service (NCARDRS) [5]. NCARDRS have expanded the systems available for follow-up of cases through administrative data and close links with the health professionals who report new diagnoses (notifiers). These changes in collection methods present a potential problem for research on trends during the transition period. It is important to have evidence that can help separate any effects of changes in data collection from changes in disease prevalence.

With England’s universal National Health Service (NHS), researchers and service planners alike are interested in establishing the potential for administrative NHS data to be used for population health monitoring and surveillance. Hospital Episode Statistics for England (HES) is a key source of hospital activity data (inpatient admissions, outpatient appointments and emergency department presentations) used for service planning and payment for hospital care funded by the NHS [6]. Linkage between registers has previously been used to assess case ascertainment (percent of cases detected) in the NDSCR and other congenital anomaly registers [4, 7], but not linkage to administrative data. Linkage between population-based registers and hospital records has been used to assess the coverage of each [8], but findings are specific to the data sources in questions.

In this article we describe the approach to linkage of NDSCR to HES and use the linked data to estimate the level of case ascertainment (proportion of all cases identified) in each data source and trends in the prevalence of Down's Syndrome among live births in England, between 1998 and 2013. In doing so, we aim to support integration of NDSCR and NCARDRS, to provide a resource for research on long-term outcomes of Down’s syndrome, and to establish methods that can be extended to linkage of NCARDRS and the full range of congenital anomaly and rare disease research that it facilitates.

## Methods

### Data sources: National Down Syndrome Cytogenetic Register

From 1989–2014, all cytogenetic laboratories in England (and Wales) notified the NDSCR of any cytogenetically confirmed diagnosis of trisomy 21 or related karyotype [9]. The register included pre- and postnatal diagnoses. Information on birth outcomes following prenatal diagnoses (live birth vs foetal death or termination) was obtained from clinicians and midwives but was missing in 8% of all diagnoses [9]. A total of 13 650 records were extracted for linkage, including 10 415 where the birth outcome was "live birth", the year of birth was between 1998 and 2013, and the postcode did not indicate residence outside of England (Figure S4, Appendix 1, Supplementary Material). A further 1226 records had missing birth outcomes but were within scope with respect to year and postcode region. The possible proportion of these that were live births is considered in the analysis, but these records had insufficient data for linkage (i.e. dates of births and NHS numbers were unknown).

### Data sources: Hospital Episode Statistics for England

With the universal healthcare provided by the NHS, HES captures 98–99% of all hospital activity in England [6]. When legally permitted and ethically justified, data are made available for research in de-personalised form (excluding names, addresses, etc.). Like most administrative data, records in HES represent events, in this case episodes of admitted patient care under one consultant, outpatient appointments, and emergency department presentations. Each patient’s records are linked through assignment of 'HESIDs' by NHS Digital, a linkage process (involving NHS number, date of birth, postcode, sex and the patient ID numbers used locally by hospitals) that is subject to linkage error [10]. Missed links lead to records belonging to the same patient being assigned different HESIDs, and false links (which are relatively rare) cause records from different patients to be assigned the same HESID [11]. The accuracy of HESIDs depends on the quality of matching data in the administrative record (NHS number, date of birth, postcode and local patient identifiers assigned by the treating hospital) which is known to be poorer in earlier years [10] and in birth episodes [12], partly because NHS numbers were not allocated at birth until after 2003 (NHS numbers were previously allocated at registration of birth or with a general practice).

Simply counting all distinct HESIDs with a Down’s syndrome diagnosis may lead to double-counting of cases when some patients have multiple HESIDs. To mitigate this, we first identified all birth episodes during our study period to identify a birth cohort which, by virtue of the fact that people are only born once, can reasonably be assumed to contain few duplicates. We then linked these to any subsequent hospital activity using HESID to identify diagnoses recorded after the birth admission. This birth cohort approach also allowed us to ensure that patients had been born in England and were therefore within the target population.

The birth cohort contained birth episodes for an estimated 10.3 million babies admitted during the 1997-98 to 2013-14 financial years. It therefore excludes babies born outside of hospital (2.2% of live births in England during 1997–2013) but represents 99.0% of the number of live births not at home recorded by the Office for National Statistics [13]. The construction of this birth cohort followed methods detailed in Harron, Gilbert [14] (Appendix 1, Supplementary Material). For each person in this cohort, Down's syndrome status was identified by the presence of any admitted patient care episode or outpatient appointment, at any time up until 2017-18 (linked by HESID), which included a three-character ICD10 "Q90" diagnosis code (i.e. including all four-character subclassifications).

While this birth cohort approach mitigates double-counting from the splitting of patient's HESIDs, there is a potential trade-off in false negative misclassification when information about Down's Syndrome diagnoses is captured after birth but cannot be linked to the birth episode (because of having separate HESIDs). We therefore compared this approach to a simple analysis of HESIDs with dates of birth within the target range (but for whom country of birth could not necessarily be confirmed). These analyses were contrasted against analysis of NDSCR records alone, and to analysis of linked data from both NDSCR and HES.

### Data linkage

**Figure 1: Linkage overview:**
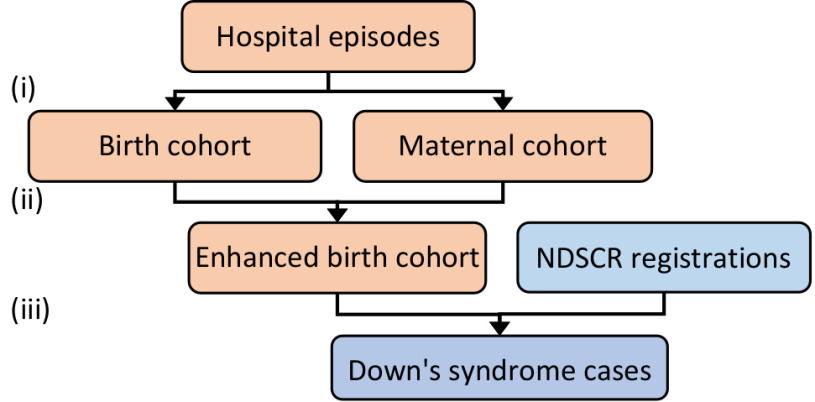


The NDSCR contains matching data relating to both the affected babies and their mothers (Table S1, Appendix 1, Supplementary Material ). In HES, however, information about babies and mothers is recorded separately. Harron and colleagues [14] demonstrated how babies can be linked to their mothers in pseudonymised HES (records with the personally identifying information usually required for linkage removed) using demographic and clinical variables captured in both admission records (mostly so-called 'baby tail' and 'maternity tail' variables). By extending their methods to the 1997-98–2013-14 financial years and incorporating additional matching data that were available at Public Health England, the birth cohort was enhanced by initial linkage of babies to their maternal delivery episodes within HES. Linkage of babies to mothers allowed maternal NHS numbers and dates of birth to be added to babies' HES records for linkage of HES to NDSCR, and for missing data in babies' postcodes to be completed using the mothers' records (Figure 1). Further details about HES cohort construction and probabilistic linkage [15] are provided in Appendix 1 (Supplementary Material).

### Estimation of prevalence and case ascertainment

Three sets of estimates of annual prevalence were generated:

1. Using NDSCR data alone, with ONS estimates of live births in England [13] as the denominator. The main analysis considered only where the birth outcome was 'live birth'. An alternative analysis that included records with unknown birth outcomes is presented in Appendix 3 (Supplementary Material).
2. Using HES data alone. The main analysis considered the proportion of the HES birth cohort to have a Down's syndrome diagnosis code recorded at any time. This analysis includes only children born in NHS hospitals in England, in both numerator and denominator. Appendix 3 (Supplementary Material) presents an alternative analysis of the proportion of all HESIDs with dates of birth within the cohort window (i.e. without requiring an identified birth episode), ever to have a Down's syndrome diagnosis code recorded. That analysis includes children born outside hospital but also outside England, in both the numerator and denominator.
3. Using linked data and capture-recapture analysis to estimate the total number of incident live birth cases, with ONS estimates of live births in England [13] as the denominator (this analysis therefore includes children born in and outside hospital, in England, in both the numerator and denominator).

**Figure 2: Subgroups for estimating prevalence and case ascertainment:**
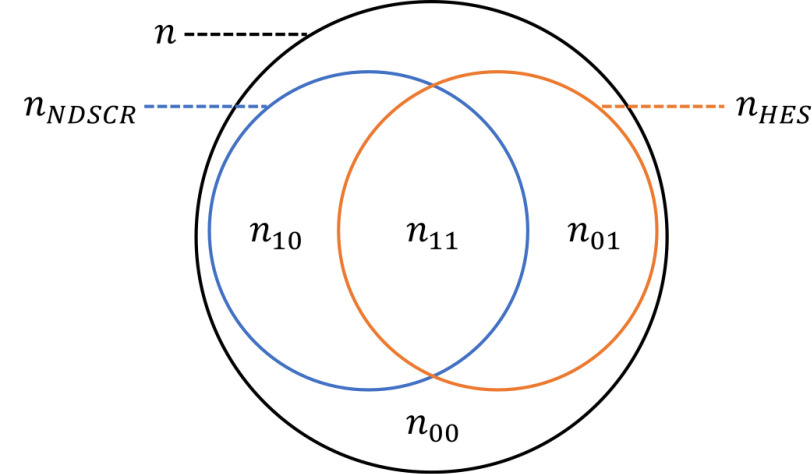


Prevalence estimation using linked data depends critically on the accuracy of linkage. If *n_NDSCR_* is the number of live birth diagnoses registered in NDSCR and *n_HES_* is the number of people with Q90 diagnosis codes in the HES birth cohort, then the total number of incident cases, n, can be divided into four key subgroups (Figure 2). Missed links between NDSCR and HES could result in somebody whose diagnosis is recorded in both sources being counted twice; once in *n*_10_ and once in *n*_01_. False links could result in two people from *n*_10_ and *n*_01_ being counted once (in *n*_11_). Capture-recapture analysis of the number of unrecorded cases (*n*_11_) relies on accurate estimation of the other subgroups [16]. We therefore assigned estimates and plausible limits for both missed links and false links, varying the assigned rates of false links with the level of evidence supporting each link (Table S2, Appendix 2, Supplementary Material ). Lastly, we also allowed for the possibility of false positives diagnostic codes to have been recorded in HES. Further details about the approach to quantitative bias analysis [17-19] and capture-recapture analysis are provided in Appendix 2 (Supplementary Material).

For each analysis, trends in prevalence were estimated using logistic regression of Down’s syndrome status on year of birth. This produced annual odds ratios that were converted into annual growth rates (% change in prevalence per year).

## Results

Results of linkage are included in Appendix 1 (Supplementary Material). Characteristics of candidate links and unlinked records are presented in Table 1 and stratified by match weight (a measure of agreement on variables used for linkage). Table S4 (Appendix 3, Supplementary Material ) provides regional statistics.

**Table 1: Characteristics of linked and unlinked records:** DOB: Date of birth HES: Hospital Episode Statistics for England MW: match weight NDSCR: National Down Syndrome Cytogenetic Register. NDSCR records exclude those with missing birth outcome. All data are column proportions, ignoring missing data, so that associations between record characteristics and linkage quality are reflected by differences in proportion across columns within each row. Probabilistic links are grouped by match weight, a score reflecting the level of agreement over matching variables (see Methods). ^1^The number of candidate links may be higher than the number of records in either file, indicating ambiguity of multiple links with equal agreement; for two of such candidate links, either at least one is false or both are true and it is the records in the contributing files that have not been completely deduplicated. Source: Hospital Episode Statistics (HES), NHS Digital (Copyright © 2019. Re-used with the permission of NHS Digital. All rights reserved) and the National Down Syndrome Cytogenetic Register (NDSCR), Public Health England.

	Deterministic links	Probabilistic (MW> 40.6)	Probabilistic (MW: 30.5–40.6)	Probabilistic (MW: 18.1–30.5)	Probabilistic (MW< 18.1)	Unlinked NDSCR records	Unlinked HES cases
n							
NDSCR records	4939	3694	449	662	534	137	-
HES records	4941	3703	446	646	654	-	2280
Candidate links1	4941	3720	454	799	739	-	-
Q90 code (in HES records)	96.4%	91.1%	70.2%	81.4%	17.0%	-	-
Difference in DOB > 180 days (in candidate links)	0.4%	< 0.3%	1.5%	3.6%	6.0%	-	-
Sex = male							
in NDSCR records	55.4%	53.9%	52.2%	52.9%	54.7%	49.3%	-
in HES records	55.1%	53.8%	52.7%	52.9%	56.3%	-	53.6%
Premature (<37 weeks)							
in NDSCR records	22.3%	19.1%	16.7%	12.7%	18.4%	10.5%	-
in HES records	23.3%	22.3%	22.5%	20.7%	10.8%	-	23.3%
Age at diagnosis (in NDSCR records)							
Prenatal	9.9%	10.0%	7.1%	3.7%	8.5%	7.4%	-
< 12 months	89.5%	89.7%	91.9%	93.7%	81.4%	85.2%	-
≥ 12 months	0.6%	0.3%	1.0%	2.6%	10.1%	7.4%	-
Age at first diagnosis code (in HES records)							
< 12 months	90.9%	89.8%	90.4%	88.2%	88.9%	-	77.7%
≥ 12 months	9.1%	10.2%	9.6%	11.8%	11.1%	-	22.3%
Number of episodes in first year of life (in HES records)							
1	22.5%	38.4%	48.6%	42.4%	78.2%	-	36.1%
2–4	42.5%	37.1%	30.4%	31.5%	15.4%	-	34.5%
≥ 5	35.0%	24.4%	20.9%	26.1%	6.5%	-	29.4%

### Prevalence and trends

Analyses of single-source data are illustrated in Figures S8 and S9 (Appendix 3, Supplementary Material). A simple analysis of HESIDs (without restriction to a birth cohort) produced prevalence estimates that were both highest and most steeply increasing, with an estimated relative annual growth rate of 1.6% (95% CI: 1.3%, 2.0%), increasing to a prevalence of 13.1 cases per 10,000 live births in 2013. Restricting HES records to a birth cohort produced results that were comparable to analysis of NDSCR data if NDSCR records with unknown birth outcomes were included, with annual growth of 1.1% (95% CI: 0.6%, 1.5%) and 0.9% (95% CI: 0.5%, 1.3%), respectively. Excluding NDSCR records with unknown birth outcomes produced the lowest and most stable prevalence estimates, increasing by 0.4% (95% CI: 0.0%, 0.8%) per year to 10.4 cases per 10 000 live births in 2013.

**Figure 3: Linkage overview:**
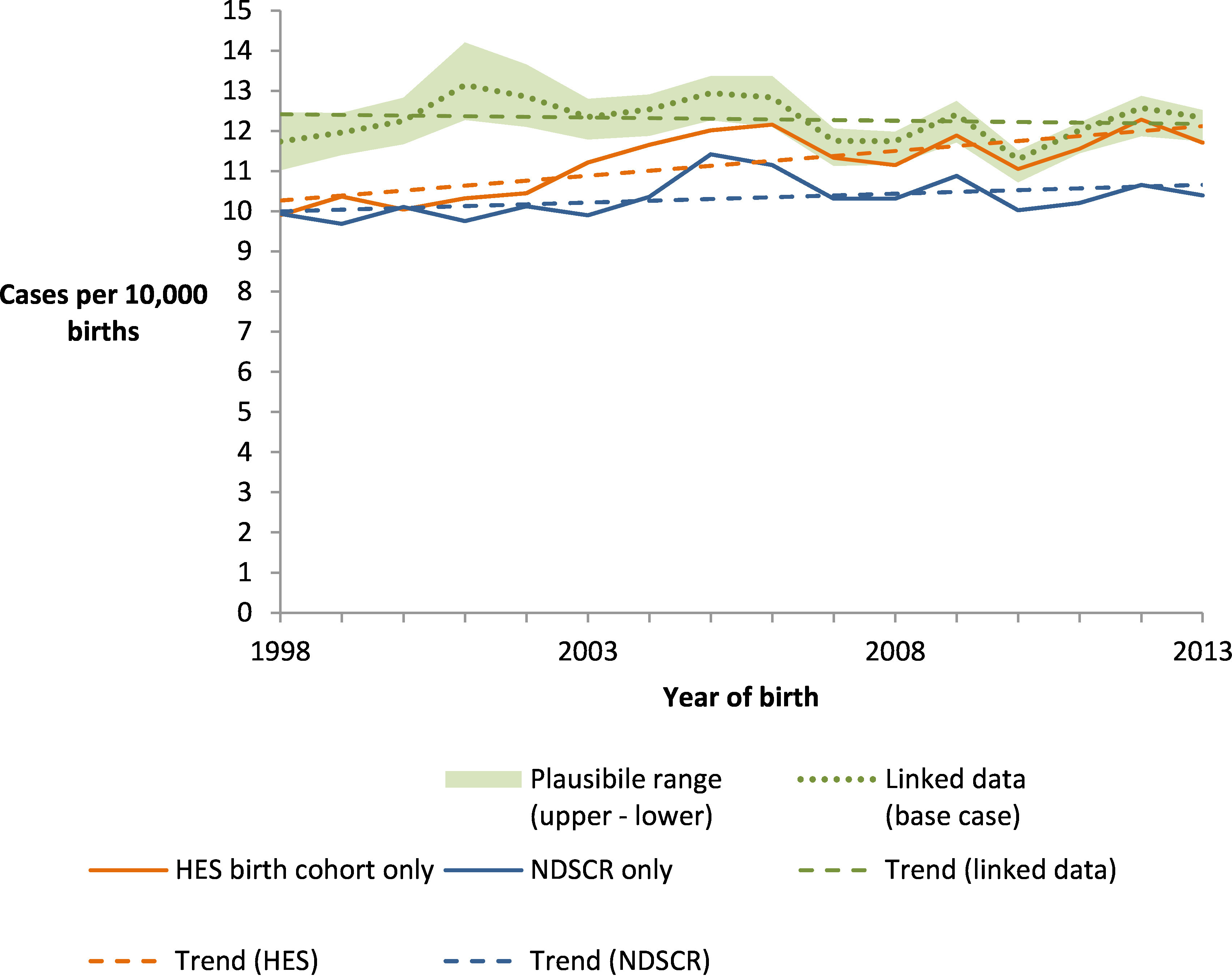
NDSCR estimates are derived using ONS births data as denominator; HES estimate is derived from the HES birth cohort data only so exclude children born outside hospital; Linked data estimates are derived using capture-recapture methods with ONS births data as denominator and quantitative bias analysis to generate plausible limits of uncertainty. Trend lines indicate fitted linear regressions of annual prevalence estimates, with slopes representing the estimated annual change in prevalence over the study period.

Source: Hospital Episode Statistics (HES), NHS Digital (Copyright © 2019. Re-used with the permission of NHS Digital. All rights reserved) and the National Down Syndrome Cytogenetic Register (NDSCR), Public Health England.

In marked contrast to these indications of increasing prevalence, linked data indicated an overall prevalence that was generally higher than single-source estimates but was stable, with no significant change over time at a 95% confidence level (estimated annual growth = -0.1% (95% CI: -0.5%, 0.2%)) and an estimated prevalence of 12.3 cases per 10 000 live births, both in 2013 and overall (Figure 3).

Quantitative bias analysis provided regions of plausibility around these base cases estimates, reflecting uncertainty in the accuracy of diagnostic codes in HES and accuracy of linkage between HES and NDSCR (Figure 3 shading). Since 2006, prevalence estimates produced by analysis of the HES birth cohort have fallen within this range. Prevalence estimates produced by NDSCR live births were consistently below this range but would have overlapped it since 2004 if records with unknown birth outcomes were included. Combining all upper and lower estimates from the quantitative bias analysis indicated a plausible range of 11.7–12.5 cases per 10 000 live births in 2013, and 11.6–12.7 overall.

**Figure 4: Linkage overview:**
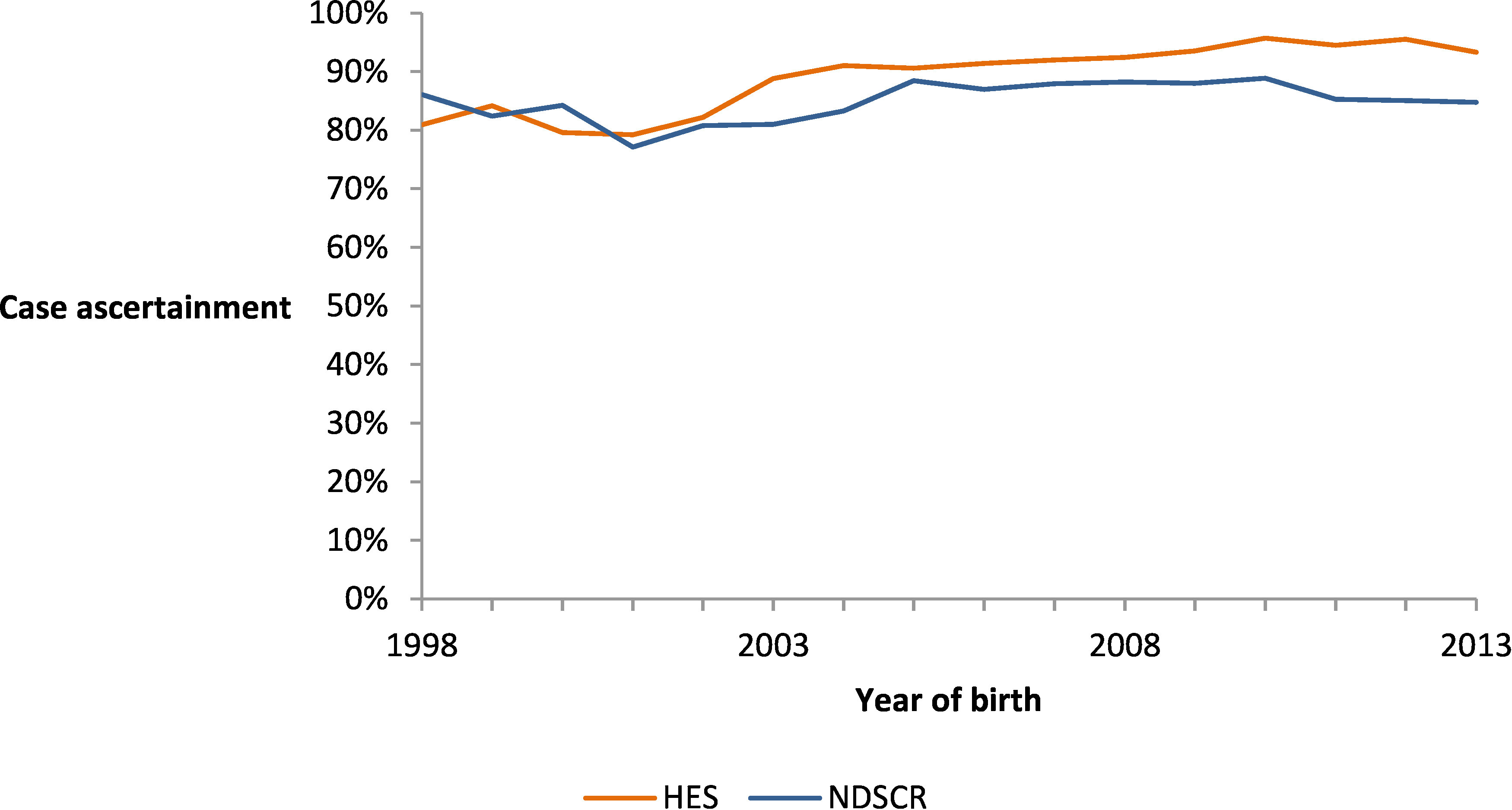
Proportions are the observed number of incident cases in each dataset (live births in NDSCR and birth cohort cases with Q90 diagnosis codes at any time in HES) divided by the base case estimated number of incident cases in England using capture-recapture analysis of linked data.

Source: Hospital Episode Statistics (HES), NHS Digital (Copyright © 2019. Re-used with the permission of NHS Digital. All rights reserved) and the National Down Syndrome Cytogenetic Register (NDSCR), Public Health England.

Subtracting the number of cases captured in single-source estimates from the number estimated in linked data indicates that case ascertainment in NDSCR varied between 74% and 88% over the study period but was more stable than in HES, which increased from 81% (1998) to 96% (2012) (Figure 4).

## Discussion

This analysis demonstrates the feasibility and value of linking a perinatal cytogenetic register to HES. Data linkage provided a picture that contrasted with both the individual data sources which, if we accept it as being less biased, illustrates how linked data can be more than the sum of its parts.

These findings highlight strengths and limitations of both data sources. Ascertainment of live births with Down’s syndrome in the NDSCR appeared lower than in HES in more recent years, possibly because of loss to follow-up of prenatal diagnoses, but NDSCR's more consistent data quality over time provided better reflection of the underlying trends in prevalence. While HES appeared to have more complete case ascertainment in recent years, changes in the quality of HES over time could have created an alarming picture of an emerging epidemic of Down’s syndrome. We propose that this can largely be explained by decreasing errors in the assignment of HESIDs, which is evidenced by the increasing recording of NHS numbers in birth episodes up until about 2009 (Figure S7, Appendix 1, Supplementary Material) and the decreasing proportion of HES cases with only one episode in their first year (Figure S10, Appendix 3, Supplementary Material). This variation in quality of administrative data over time can distort analysis of trends and could confound evaluation of changes in policy or universal health care services, such as the recent introduction of free non-invasive screening for Down’s syndrome.

The problem of missed links in the assignment of HESIDs is poorly documented and there is little information available to help analysts address it. It is also statistically complex; in the simple analysis of all HESIDs it is likely to have contributed to overestimation from double-counting (splitting of one patient’s records into multiple observational units) while, in analysis of the birth cohort, it is likely to have contributed to underestimation through false negative misclassification of patients with missed links to their subsequent HES records that contain diagnosis codes [17]. Like many administrative datasets, HES is generated by service events that must be linked before person-level analyses can be implemented. With missed links in person identifiers having potential to wreak such statistical havoc, they are an issue that requires focused mitigation efforts by data providers through linkage quality assessment [20] and by researchers through sensitivity and bias analysis [17-19].

With HES providing such a critical resource for health services research, and birth episodes containing unique information about perinatal health and family characteristics, there is a strong argument for refining the HESID algorithm to better support linkage of birth episodes by incorporating and leveraging familial links. While mother-child linkage is possible with the current data, a more complete record of familial links could be constructed with routine linkage to other health data, such as general practice registrations and routine child health checks.

Of course, the linked dataset is not a 'gold standard' and is itself prone to error. There are many possible sources of error and bias in this analysis, but we have attempted to quantify the main ones. People born earlier in the cohort had a longer period of observation in which HES events could be captured, but in the linked data this was accounted for by the capture-recapture analysis. More sophisticated methods for extracting diagnostic information from HES, using related diagnoses, procedure codes or outpatient appointments, may result in improved case ascertainment. Improvements in linkage might also be possible if NDSCR records with missing birth outcomes are considered, with approximate matching between date of sample and date of birth (sample date was not available for this linkage).

The main unaccounted potential source of error is in the assumptions of the capture-recapture analysis. Aside from no linkage error, the three main assumptions of the formula used are (i) equivalent source populations, (ii) homogeneity in probabilities of detection and (iii) independence of the data sources [16]. Most concerning is the source populations, which are known to have slight differences; people not born in hospital (an estimate 3% of all births) have no opportunity for their Down's status to be 'captured' in the HES birth cohort. By increasing the number of people identified only by NDSCR, this is likely to have led to overestimation of the number of unrecorded cases (cases detected by neither source), and therefore overestimation of the total prevalence and underestimation of case ascertainment in each dataset (the denominator for case ascertainment is the estimated total prevalence). We considered accounting for this quantitatively but this became complicated by implausible *combinations* with other bias parameters, suggesting that the potential for bias was already encompassed within the plausible range.

Heterogeneity in the probability of detection in either data source could have occurred if some parts of the population were both less likely to be screened and less likely to be born in hospital (e.g. people from rural and remote areas). This could similarly have inflated linked data estimates but may have been offset by dependence between the data source, if people recorded in one data source were more likely to be recorded in the other (e.g. because of related data collection mechanisms).

Regardless of these three assumptions, the contribution of unrecorded cases (those estimated through capture-recapture) was relatively small at between 0.8% (2012) and 6.2% (2001) (Table S3, Appendix 2, Supplementary Material). Even if these phenomena varied over time—which there is no obvious reason to suspect—they could not feasibly have accounted for all of the observed differences in trends.

In 2014, the NDSCR was integrated into the National Congenital Anomaly and Rare Disease Registration Service (NCARDRS). NCARDRS now integrates information from every maternity unit in England, has established electronic data feeds from cytogenetic laboratories, and is able to trace birth outcomes through the NHS Summary Care Record. Our findings about live births recorded in the NDSCR are therefore unlikely to be generalisable to NCARDRS, while data for all registrations (i.e. including unknown birth outcomes) may be more comparable.

Similarly, our findings with respect to identification of Down's syndrome in HES cannot be generalised to other diseases, phenotypes or datasets (see [8] for a relevant example with contrasting findings). There is considerable variation in how diagnostic information is recorded in administrative data across diagnoses, between datasets and potentially over time, so it is important to assess the quality of administrative data sources in the context of each analysis.

When interpreting trends in live birth prevalence of Down's syndrome internationally, authors typically focus on the disentangling the competing effects of increasing risk factors for Down's syndrome (e.g. maternal age) and increasing rates of termination [21, 22]. Using surveillance data, de Graaf and colleagues [23] estimate an increasing trend in live birth prevalence in the US over a similar time period. Using hospital records, the Public Health Agency of Canada [24] estimate a stable trend. Ours is the only known example to have used data linkage to control for quality issues in the underlying data sources.

## Conclusion

The differing conclusions that could be drawn from linked data versus single data sources highlight both the value that data linkage can offer and the dangers that it can pose when the quality of linkage is ignored. Reassuringly, we demonstrate that even when the quality of matching data is poor and there is uncertainty in linkage, quantitative bias analysis can be used to identify plausible boundaries within which target parameters should lie [17]. Probabilistic techniques [18] could further enhance quantification of this uncertainty.

When NDSCR and HES were linked, we found that detection of live birth cases in NDSCR increased over time, resulting in a slowly increasing trend in live birth prevalence of Down's syndrome. In HES, we observed a potentially alarming increase in prevalence that appeared partly attributable to internal linkage errors in the assignment of HESIDs. In the linked data, the trend appeared contrastingly stable. Given the value that this study demonstrates in linking registry with administrative data, the fairest basis for analysis of trends during and after transition from NDSCR to NCARDRS is likely to be provided by integrating NDSCR-HES linked data with a future linkage of NCARDRS to HES. Such routine linkage of registry and administrative data can provide other benefits also, in this case including invaluable support for analysis of long-term health outcomes.

## Ethics statement

 Linkage between NDSCR and HES was conducted to facilitate the research project ‘Evaluating variation in special educational needs provision for children with Down syndrome and associations with emergency use of hospital care’, with approval from the Health Research Authority’s London – Camden and King’s Cross Research Ethics Committee (ref: 16/LO/0094) and Confidentiality Advisory Group (ref: 16/CAG/0015), and the Administrative Data Research Network (ref: PROJ-165).

## Supplementary Material

Appendix

## Abbreviations

HES	Hospital Episode Statistics for England (NHS Digital)
HESID	A unique patient pseudo-identifier assigned by NHS Digital to identify hospital episode records that relate to the same person
NCARDRS	National Congenital Anomaly and Rare Disease Registration Service (Public Health England)
NDSCR	National Down Syndrome Cytogenetic Register (Public Health England)
NHS	National Health Service
NHS Digital	Trading name of the Health and Social Care Information Centre
