# Supplementary material for: Prevalence of Down's Syndrome in England, 1998–2013: Comparison of linked surveillance data and electronic health records
Source: Int J Popul Data Sci. 2019 Mar 19;5(1):1157. doi: 10.23889/ijpds.v5i1.1157 (PMC7115985; doi:10.23889/ijpds.v5i1.1157)
Supplement: Appendix [file ijpds-05-1157-s001.pdf]

Doidge, JC et al. Prevalence of Down's Syndrome in England, 1998–2013: Comparison of linked surveillance data and electronic health records
https://doi.org/10.23889/ijpds.v5i1.1157

Appendix 1: Methods for cohort construction and linkage

Construction of cohorts of babies and mothers in Hospital Episode Statistics for England

The unit of recording in the Admitted Patient Care section of HES is an episode of care under one consultant. An admission may be comprised of multiple episodes and patients may have multiple admissions over time, plus attendances in outpatient clinics and emergency departments. These are linked by NHS Digital, who assign each record a 'HESID' indicating a distinct patient. Being a linkage procedure, allocation of HESIDs is subject to linkage error; missed links that result in people's records being allocated different HESIDs and false links that result in different people sharing one HESID. There has been little evaluation of the algorithm used to assign HESIDs but previous experience highlighted increased error rates in birth episodes, stemming from the allocation of NHS numbers after birth registration (i.e. *after* discharge from birth admissions) and a known error in recording of infants' postcodes prior to 2011 [1, 2].

To mitigate errors in HESID, we adopted methods for combining episodes relating to the same person that did not rely solely on HESID, based on those described in Harron, Gilbert [1]. On extending these methods to the 1997-98 to 2001-02 years, changes in the way that baby/maternity tail variables were recorded during the earlier years meant that additional criteria had to be incorporated. The processes for constructing the birth cohort is summarised in Figure S1.

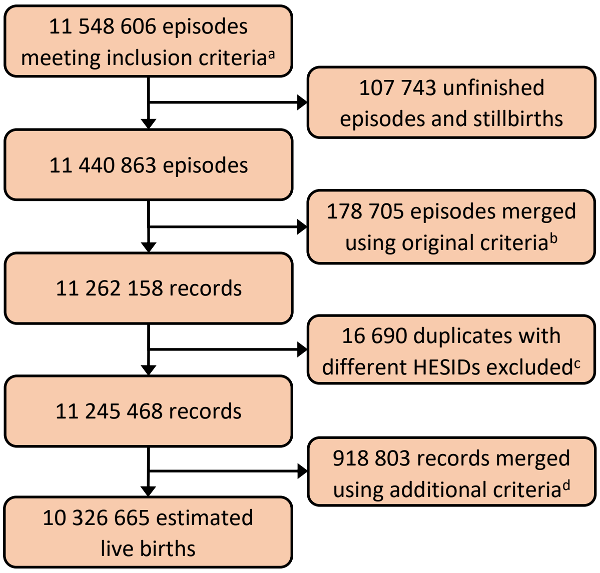

Figure S1 Construction of the HES birth cohort

[a] As per Appendix S1 of Harron and colleagues [1].
[b] As per Harron and colleagues [1]: Match on HESID, start date, age, postcode, birth order and birth weight.
[c] As per Harron and colleagues [1]: Match on start date, age, hospital, GP practice, ethnicity, date of birth, and baby tail field but with different HESIDs.
[d] No more than two out of 21 potential inconsistencies on demographic and baby tail variables within HESID.
Source: Hospital Episode Statistics (HES), NHS Digital (Copyright © 2019. Re-used with the permission of NHS Digital. All rights reserved)

Similarly, additional criteria had to be incorporated into the processing of maternal records (Figure S2). This reflected both changes in the way maternity tail variables were recorded over time and the additional requirement in this application to link data for multiple births. Each maternal admission record that indicated a multiple birth was reshaped to create a separate record for each baby, and additional exclusion rules then applied to address the large volumes of invalid/not applicable codes contained in the multiple birth fields (the maternity tail variables indexed by "_[N]") required additional exclusion criteria to be applied to these reshaped maternal records (Figure S2, footnote (d)).

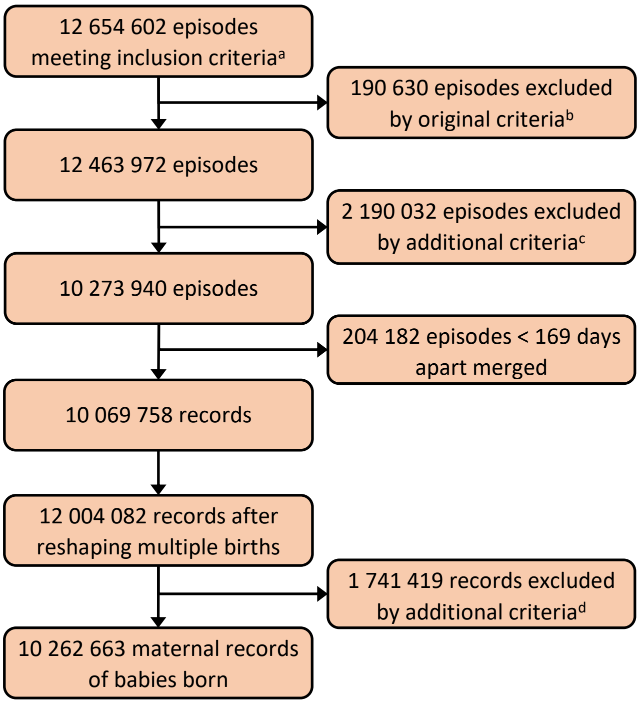

Figure S2 Construction of the maternal cohort

[a] As per Appendix S1 of Harron and colleagues [1].

[b] As per Appendix S1 of Harron and colleagues [1].

[c] Less than 2 valid maternity codes after cleaning *and* no relevant procedure code *and* no relevant diagnostic code in the first five diagnosis fields.

[d] Birth characteristics relating to different babies in multiple births are indexed by _[N] (e.g. birthweight_1 birthweight_2, etc) which becomes a variable when these data are reshaped. The field 'NUMBABY' separately records the number of babies associated with a delivery. Reshaped records were excluded whenever: (index > 3) or (index = 3 and NUMBABY = 1) or (index = 2 or 3, and the number of babies indicated by reshaping was greater than 5, and NUMBABY = 1 or more than 5).

Source: Hospital Episode Statistics (HES), NHS Digital (Copyright © 2019. Re-used with the permission of NHS Digital. All rights reserved)

Comparison of the cohort sizes to estimates of live births in hospitals generated from birth registration records by the Office for National Statistics suggest that any double-counting arising from the allocation of multiple HESIDs to single patients within the birth cohort is minimal (Figure S3).

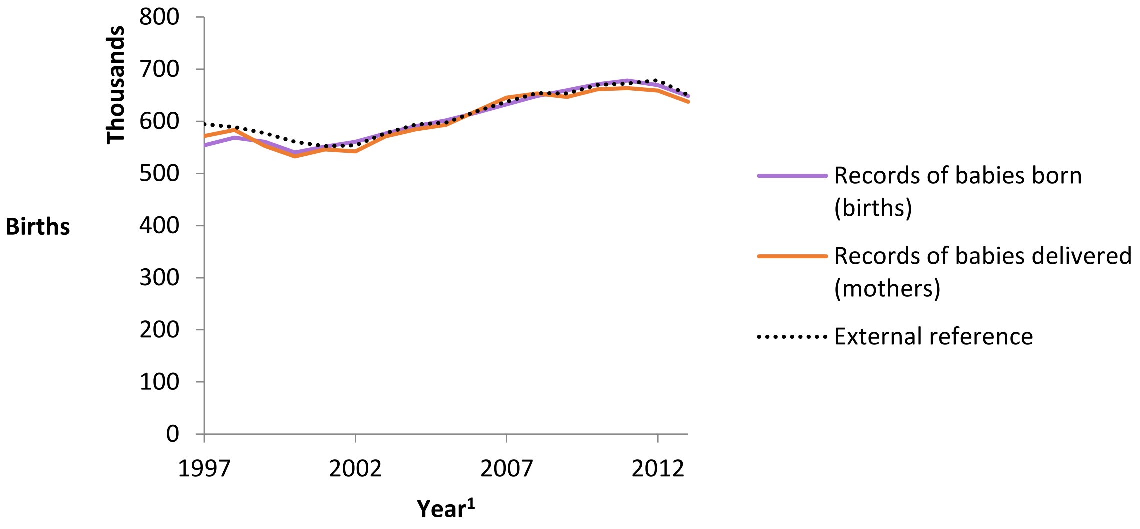

Figure S3 Comparison of cohort sizes to ONS estimates of births in hospital in England
External reference data was births not at home, derived from birth registration data by
[1]ONS data are calendar years; HES data are financial years commencing, so some difference is expected
Source: Hospital Episode Statistics (HES), NHS Digital (Copyright © 2019. Re-used with the permission of NHS Digital. All rights reserved) and the Office for National Statistics [3].

Selection of records from the National Down Syndrome Cytogenetic Register

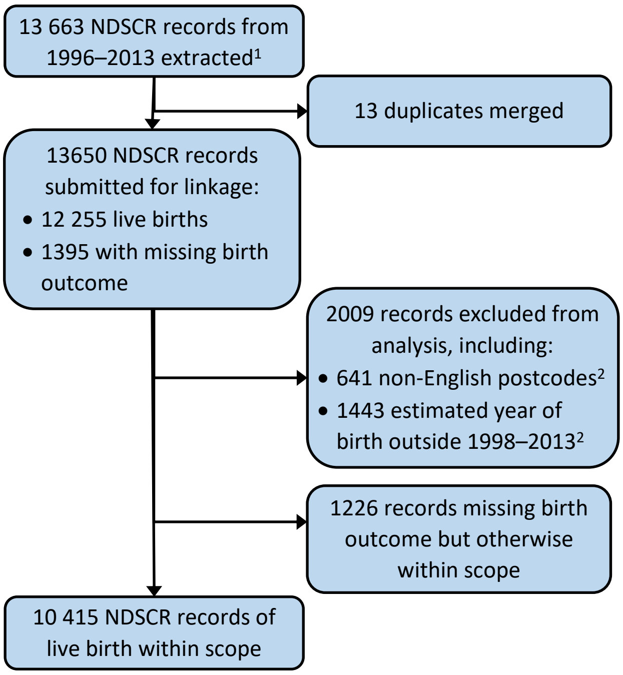

Figure S4 Selection of records from the National Down Syndrome Cytogenetic Register (NDSCR)
[1] Extraction and linkage initially included records with year of birth = 1997 or year of sample = 1996 for prenatal diagnoses. Because HES records for 1997 were not available for the full calendar year, this analysis excludes these records.
[2] Including 75 records with both criteria

Linkage of babies to mothers in Hospital Episode Statistics for England

The linkage implemented by Harron and colleagues [1] involved 23 'pseudonymised' matching variables; mostly clinical fields contained in the baby and maternity tails, plus postcode district (derived by NHS Digital from HOMEADD), mother's age (MATAGE, derived by NHS Digital from the mother's and baby's dates of birth) and an estimated date of birth for the baby (derived by the authors from the date of procedure or admission). Our linkage was additionally supported by access to full postcodes and dates of birth. Postcodes for babies, if missing, were imputed (carried backwards) from the first non-missing admission or outpatient appointment for that HESID.

As with Harron and colleagues [1] we used a two-step linkage procedure involving an initial deterministic step using a subset of matching variables that uniquely identify some individuals, and a probabilistic step that used all matching variables. The deterministic step provided a reference set for estimation of m values (the probability that a matching variable agrees if the records are a match) for use in calculation of match weights in the probabilistic step. Our deterministic step combined two rules:

1. Unique agreement on financial year, hospital trust, general practice, sex, birth order, gestational age and mother's age, with no disagreement on infant's date of birth or mother's date of birth, *or*
2. Unique agreement on financial year, hospital trust, infant's date of birth and mother's date of birth, with no disagreement on general practice, sex, birth order or mother's age

Use of variables in the deterministic step precludes estimating their m values using this data. For variables used in deterministic linkage, m values were informed by previous implementation [1]. m values for the remaining matching variables were estimated as the proportion of deterministic links exhibiting agreement on each matching variable. u values were estimated using random draws and were value-specific where possible. For each pair of records, partial match weights were summed across all matching variables to calculate an overall match weight, assigning partial weights of zero in the presence of missing values.

The probabilistic step only considered records that matched on hospital trust and in which the baby's admission commenced no more than seven days before the mother's admission commenced and no more than seven days after the mother's admission ended. Candidate links were ranked by match weight and sorted into 'unambiguous links' in which the top-ranked infant record for a maternal record and the top-ranked maternal record for an infant record were consistent and uncontended, multiple links in which the top rank was shared by multiple candidates (which could all be true, given potential linkage errors in the construction of the cohorts) and 'ambiguous links' in which the highest ranks were inconsistent. An iterative sample of record pairs was clerically reviewed to select a minimum threshold for accepting these candidate links (in this case, match weight ≥ 3). In summary, the probabilistic linkage steps were to:

1. Identify mother and baby records from the same hospital that are no more than seven days apart in time.
2. Calculate match weights and rank all candidate links by match weight
3. Use iterative, sampled clerical review to decide a minimum match weight for accepting links.

4. Accept unambiguous links above this threshold, where the highest ranked baby for a mother is the same as the highest ranked mother for that baby.
5. Flag links above the threshold where there is inconsistency the highest ranked pairs as potential errors in linkage.
6. Flag links above the threshold where there is ambiguity in the highest ranked pairs as potential errors in linkage or true multiple links (reflecting multiple records for the same mother or baby in the data).

Overall, 49.7% of infants in the birth cohort were able to be linked deterministically to a mother and, for a further 44.0%, an unambiguous probabilistic link was identified. Small numbers of multiple (0.5%) and ambiguous (1.5%) links were found, with 4.3% of births remaining unlinked (Figure S5). Linkage of baby to mother was slightly less likely if the HES record indicated Down's syndrome (88.9% of birth cohort members with any Q90 diagnosis codes were able to be linked to a maternal record, compared with 95.7% of birth cohort members without Q90 diagnosis codes). Babies with no linked maternal record had fewer variables on which to link with NDSCR.

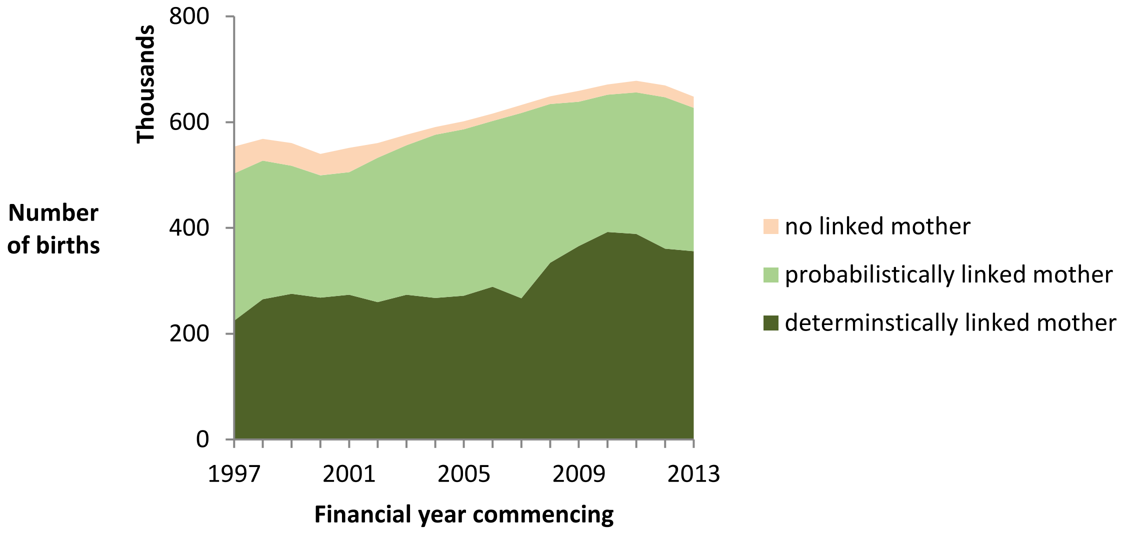

Figure S5 Linkage of babies to mothers in Hospital Episode Statistics for England, by financial year

Source: Hospital Episode Statistics (HES), NHS Digital (Copyright © 2019. Re-used with the permission of NHS Digital. All rights reserved).

Linkage of enhanced HES birth cohort to NDSCR

After enhancing the birth cohort with matching variables from their linked mothers, potential matching variables available for the HES-NDSCR linkage included: NHS numbers for both mother and child, dates of birth for both mother and child (each split into day, month and year to accommodate partial entries in NDSCR), postcode (split into two parts to accommodate partial entries), sex, gestational age, birth weight, multiple birth status, and Down's Syndrome status (constant in NDSCR, and as indicated by diagnosis codes in HES).

Linkage involved an initial deterministic (rule-based) step, which supported a subsequent probabilistic step, involving match weights (scores, based on the Fellegi and Sunter [4] framework). The deterministic linkage used child's NHS number with clerical review of all

returned links that disagreed on other matching variables. The identified links were then used to estimate m values (the proportions of true links that agree on each matching variable) for the remaining matching variables, and u values (the proportions of true non-links that agree on each matching variable) were estimated using a random draw. These m and u values were used to construct match weights, that were used to rank candidate HES links for each NDSCR record, to identify the most likely candidates. The highest ranking candidate HES record for each NDSCR record was retained and stratified according to match weight, indicating the degree of correspondence between the records. Estimated m and u values and their corresponding partial match weights are provided in Table S1, and completeness of matching variables is illustrated in Figure S6 and Figure S7.

Table S1 Match weights in HES-NDSCR linkage

Matching variable	m^1	u^2	Partial weight if agree[3]	Partial weight if disagree[4]
Day of birth	0.99	0.03	4.92	-6.09
Month of Birth	0.99	0.08	3.58	-7.50
Year of Birth	1.00	0.06	4.17	-8.28
Sex	0.99	0.50	0.98	-5.86
Birth weight	0.90	2.89E-03	8.27	-3.26
Gestational age	0.77	0.14	2.49	-1.91
Multiple birth flag	0.99	0.92	0.11	-2.86
Down's syndrome status	0.96	1.16E-03	9.69	-4.79
Postcode (first part)	0.95	7.30E-04	10.35	-4.43
Postcode (second part)	0.89	3.17E-04	11.46	-3.22
Mother's NHS number	0.96	1.00E-06	19.87	-4.58
Mother's day of birth	0.97	0.03	4.90	-5.23
Mother's month of birth	0.97	0.08	3.54	-5.13
Mother's year of birth	0.98	0.03	4.97	-5.65

[1]Estimated proportion of (true) matches exhibiting agreement on matching variable, if not missing, derived from observed proportion among deterministic links.

[2]Estimated proportion of (true) non-matches exhibiting agreement on matching variable, derived from random sample of all record pairs.

[3] $log_2(\frac{m}{u})$

[4] $log_2(\frac{1-m}{1-u})$

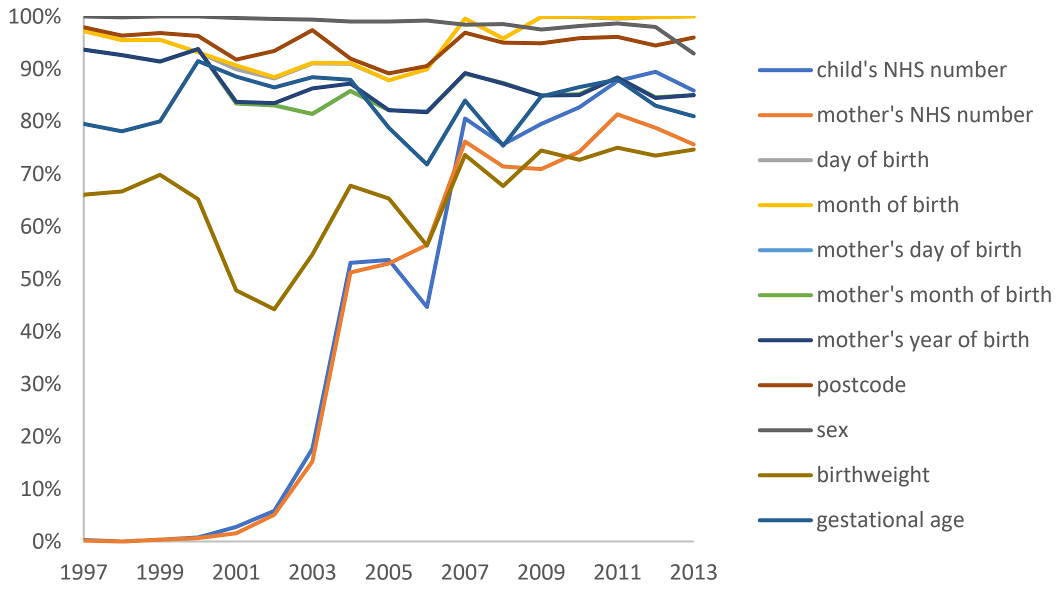

Figure S6 Completeness of NDSCR matching variables

Source: National Down Syndrome Cytogenetic Register (NDSCR), Public Health England.

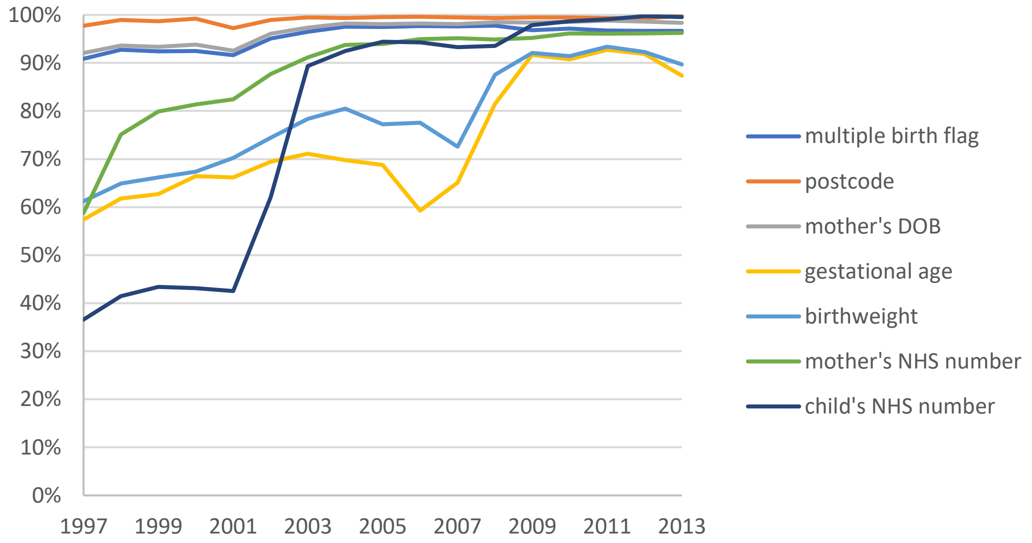

Figure S7 Completeness of HES matching variables (for HES-NDSCR linkage)

Child's date of birth, and Down's Syndrome status (as indicated by diagnosis codes) were complete in all years

Source: Hospital Episode Statistics (HES), NHS Digital (Copyright © 2019. Re-used with the permission of NHS Digital. All rights reserved).

Because NHS numbers were largely missing in both HES and NDSCR prior to 2003 (Figures S5 and S6, Supplementary Appendix 1), linkage between NDSCR and HES relied mostly on probabilistic techniques in these years. NHS numbers were also entirely missing in the prenatal NDSCR records with missing birth outcome. Of the NDSCR records of live births that were within scope, 4939 (47.4%) were linked deterministically to members of the HES birth cohort, 96.4% of whom also had Q90 diagnosis codes (see Table 1 in main article). The existence of two deterministic links for each of two NDSCR records indicated a very small degree of residual double-counting in the HES birth cohort (instances where the same person was probably represented twice; 0.04% of deterministically linked records, but

potentially higher among others). Probabilistic linkage identified candidate links for another 5339 (51.3%) live birth NDSCR records.

Of the deterministic links and probabilistic links with match weights greater than 18.1, most had Down's syndrome diagnosis codes. Of the probabilistic links with match weights below 18.1, few had diagnosis codes but this is to be expected given the contribution of diagnosis codes towards match weights (Table S1). Female records were slightly less likely to be linked, as were postnatal diagnoses, especially those occurring after 12 months of age. HES cases with multiple admission episodes in their first year of life were also more likely to be linked to NDSCR than those with only a single episode.

Appendix 1 References

1. Harron K, Gilbert R, Cromwell D, van der Meulen J. Linking Data for Mothers and Babies in De-Identified Electronic Health Data. PLoS One. 2016;11(10):e0164667. doi: 10.1371/journal.pone.0164667.
2. Hagger-Johnson G, Harron K, Fleming T, Gilbert R, Goldstein H, Landy R, et al. Data linkage errors in hospital administrative data when applying a pseudonymisation algorithm to paediatric intensive care records. BMJ Open. 2015;5(8):e008118. doi: 10.1136/bmjopen-2015-008118.
3. Office for National Statistics. Number of live births at home and total live births, England, 1994 to 2014 birth registrations. 2016.
4. Fellegi I, Sunter A. A theory for record linkage. J Am Stat Assoc. 1969;64:51-79. doi: 10.1080/01621459.1969.10501049.

Appendix 2: Methods for quantitative bias analysis and capture-recapture analysis

This appendix provides additional explanation of the quantitative bias analysis and capture-recapture analysis. Bias parameters (rates of different types of error and their plausible limits) were estimated through author consensus, then combined with capture-recapture methods [1] to estimate the number of unrecorded cases and total incident live births with Down's syndrome in England. The assigned bias parameters are summarised in Table S2 and stepped results are summarised in Table S3.

Potential false positive diagnostic codes in HES

Because it was derived from cytogenetic laboratories, we assumed that NDSCR records would not include false positive *diagnoses* (records with unknown birth outcomes may not all have been liveborn, but these were excluded from analysis). For HES, we evaluated the positive predictive value of Q90 diagnosis codes (PPV; the proportion of records with codes that truly have Down's syndrome) by examining the proportion of cases where only a single code was recorded despite many records existing for that HESID. For all cohort HESIDs that had a Q90 diagnosis code in any record, there were a total of 132,855 admitted patient care episodes. Of these, 95,593 (70.5%) included a Q90 code. By restricting this to HESIDs with at least 5 or 10 episodes (7784 and 4316 HESIDs, respectively), we could see that 95.3% (7419) and 95.5% (4121) had at least two episodes containing a diagnosis code. It seems reasonable to assume that the PPV for *multiple* Q90 codes is approximately 100%. These statistics therefore support a minimum plausible limit of 95.0% for the PPV of having *any* Q90 code and a true PPV that is likely higher. For the analysis of linked data, we assigned a base case PPV of 99.5% to having *any* Q90 code, with plausible limits of 95.0–100.0%

Estimates of linkage error

Because all deterministic links were based on unique identifiers and all deterministic links with high levels of disagreement on other matching variables had been clerically reviewed (with any questionable links subjected to probabilistic linkage instead), we assumed that the precision of linkage (proportion of links that are true) was 100% for these. For probabilistic links, we assigned point estimates and plausible limits for precision that decreased with match weight from 99–100% above a match weight of 40.6, down to between 50–100% at match weights between 0.0 and 18.1 (Table S). For the proportion of unlinked records that were in truth missed links (cases that truly appear in both datasets but for which a link could not be identified), we had little to base estimates on so assigned wide plausible limits of 10–90%. For example, if there were 50 unlinked NDSCR records and 100 unlinked HES cases in a given year, then the maximum possible number of links between these was 50 and we estimated that between 5 (10%) and 45 (90%) of these were missed links.

Capture-recapture analysis

From the basic formula for capture-recapture analysis, the number of unrecorded cases can be given by the formula $n_{00} = n_{NDSCR} n_{HES} / n_{11}$ [1]. Other than an absence of linkage error, there are three further assumptions of this formula: (i) that the data sources are independent, (ii) that cases are homogenous (equal) in terms of their probability of detection and (iii) that the population sampled by each data source is identical. In applications of capture-recapture to disease surveillance, these assumptions are often not met [2]. We therefore liaised with data collectors to qualitatively assess each assumption's plausibility and potential implications (see Discussion).

Formulae for quantitative bias analysis and capture-recapture analysis

1. Analysis of linked data without accounting for any potential sources of error
   For each year, calculate the number of cases as:
   $$\begin{aligned}Total\ cases &= NDSCR\ live\ births\\ &+ HES\ live\ births\ with\ Q90\ diagnosis\ codes\ but\ no\ link\ to\ NDSCR\end{aligned}$$
2. Adjust for **false positive diagnoses** in HES
   For each scenario (upper, base case, lower) and each year, estimate the total number of cases correctly positively identified by Q90 diagnosis codes in the HES birth cohort as:
   $$true\ diagnoses, n_{HES} = observed\ diagnoses - observed\ diagnoses \times (1 - PPV)$$
   where PPV is the positive predictive value for Down's syndrome status given the presence of at least one Q90 diagnosis code at any time (a defined bias parameter).

3. Estimate number of true matches (record pairs that should link), adjusting for **false links**
   For each category of links (deterministic plus four categories of probabilistic), each scenario, and each year, calculate:
   $$\begin{aligned}&estimated\ number\ of\ matches, given\ false\ links\\ &\quad = maximum\ observed\ links \times estimated\ precision\end{aligned}$$
   where $maximum\ observed\ links$ is the lower of the number of NDSCR records or HES records that contribute to a set of identified candidate links (i.e. assume one-to-one linkage, so that for 10 records from file A and 20 records from file B that form a set of candidate links, there can be at most 10 links in that set), and $precision$ is the proportion of links that are true matches (the positive predictive value of linkage).

4. Estimate number of true matches (record pairs that should link), adjusting for false links and **missed links**
   For each scenario and each year, calculate:
   $$\begin{aligned}&estimated\ number\ of\ matches, given\ false\ links\ and\ missed\ links\\ &\quad = estimated\ number\ of\ matches, given\ false\ links + \alpha\\ &\quad \times estimated\ number\ of unlinked\ records\end{aligned}$$
   where α is the estimated proportion of unlinked records that are missed links (a defined bias parameter, relating to the sensitivity or recall of linkage) and the number of unlinked records is taken from the lower of the estimated number of unlinked NDSCR records or unlinked HES records with diagnosis codes, after combining the estimated number of false links from Step 2 with the number of each that have no candidate links in each dataset.

5. Estimate number of true Q90 diagnoses in HES that have true matches in NDSCR, adjusting for **false links**
   For each scenario and each year, repeat Step 2 using the estimated subset of HES records that have true Q90 diagnosis codes from Step 1 (for simplicity, in this step we

assumed that false positive diagnoses were concentrated among the unlinked HES records with diagnosis codes, so that HES records with both diagnosis codes and links were assumed to be true Q90 diagnoses but not necessarily true links).

6. Estimate number of true Q90 diagnoses in HES that have true matches in NDSCR adjusting for false links and **missed links** (n_{11})
For each scenario and each year, repeat Step 3 using the estimated number of matches from Step 4, the estimated number of unlinked Q90 diagnoses in HES implied by Step 4, and the same estimated number of unlinked NDSCR records implied by Step 2 and used in Step 3.

7. Estimate total cases, including **unrecorded cases**
For each year (and each of the scenarios produced above) calculate:
$$estimated\ number\ of\ cases, n = \frac{n_{NDSCR} \cdot n_{HES}}{n_{11}}$$
where n_{NDSCR} is the number of live birth diagnoses registered in NDSCR, n_{HES} is the estimated number of live births with true Q90 diagnoses in the HES birth cohort (from Step 1), and n_{11} is the estimated number true matches between these (from Step 5).
    a. Unrecorded cases can now be derived as:
$$n_{00} = n + n_{11} - n_{NDSCR} - n_{HES}$$
    b. And case ascertainment can now be derived as $\frac{n_{NDSCR}}{n}$ and $\frac{n_{HES}}{n}$

Table S2 Bias parameter estimates used in analysis of linked data

Bias parameter	Analysis[a]		
	Lower limit	Base case	Upper limit
**Linkage precision, by link quality**			
Deterministic	100.0%	100.0%	100.0%
Probabilistic (match weight > 40.6)	100.0%	100.0%	99.0%
Probabilistic (match weight: 30.5-40.6)	100.0%	98.0%	95.0%
Probabilistic (match weight: 18.1-30.5)	100.0%	90.0%	80.0%
Probabilistic (match weight < 18.1)	100.0%	80.0%	50.0%
**Proportion of unlinked records that are missed links**	90.0%	50.0%	10.0%
**Positive predictive value of diagnosis codes among unlinked HES cases**	95.0%	99.5%	100.0%

[a]Estimates and plausible limits were assigned by author consensus (see text for further explanation). Limits are arranged such that lower limits of each parameter translate into the lowest estimates of prevalence using linked data.

Table S3 Estimated incidence of Down's Syndome, sequentially adjusted for each source of possible error

Year	Estimated number of cases, by analysis step						
	Live birth diagnoses in NDSCR	Live births with Q90 codes in HES birth cohort	Accepting maximum number of candidate links	Adjusted for false positive diagnoses [a]	…and adjusted for false links [a]	…and adjusted for missed links [a]	…and adjusted for undetected cases [a]
1998	598	562	696	693	701	684	707
1999	571	583	689	686	705	681	705
2000	579	547	684	681	702	674	702
2001	550	565	712	709	741	695	741
2002	573	583	707	704	727	696	727
2003	584	640	716	713	728	710	728
2004	629	687	757	754	761	748	761
2005	700	717	793	789	794	784	794
2006	709	745	819	815	816	806	816
2007	676	707	774	770	770	762	770
2008	694	727	790	786	790	782	790
2009	730	776	837	833	833	825	833
2010	689	742	779	775	776	772	776
2011	702	778	825	821	827	819	827
2012	740	831	880	876	873	866	873
2013	691	761	819	815	819	810	819

[a] Base case estimates, rounded to nearest integer

Appendix 2 References

1. Stephen C. Capture-Recapture Methods in Epidemiological Studies. *Infect Control Hosp Epidemiol* 1996; **17**: 262-6.
2. Braeye T, Verheagen J, Mignon A, et al. Capture-Recapture Estimators in Epidemiology with Applications to Pertussis and Pneumococcal Invasive Disease Surveillance. *PLoS One* 2016; **11**: e0159832.

Appendix 3: Supplementary results

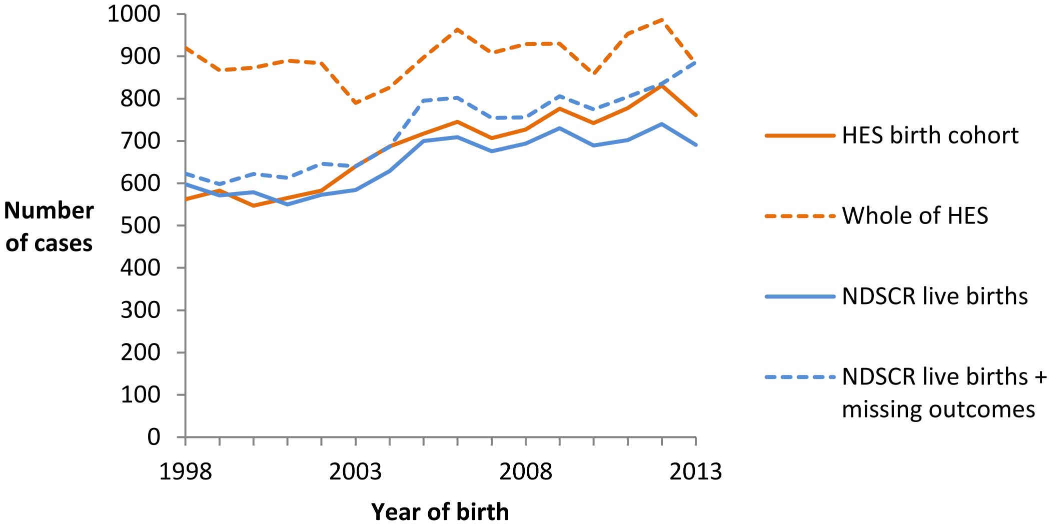

Figure S8 Annual number of Down's Syndrome cases detected in separate data sources
HES: Hospital Episode Statistics for England; NDSCR: National Down Syndrome Cytogenetic Register
Source: Hospital Episode Statistics (HES), NHS Digital (Copyright © 2019. Re-used with the permission of NHS Digital. All rights reserved) and the National Down Syndrome Cytogenetic Register (NDSCR), Public Health England.

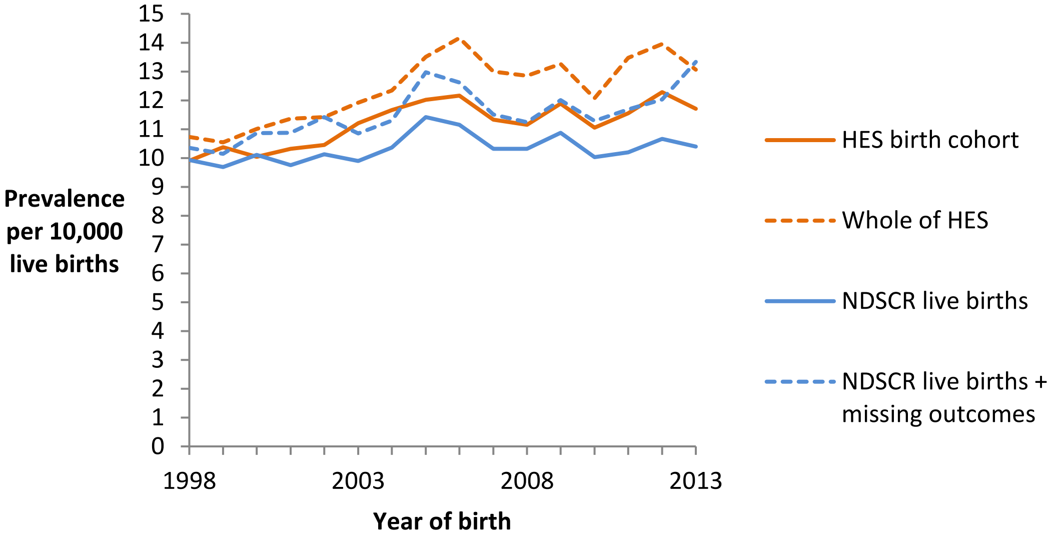

Figure S9 Annual prevalence of Down's Syndrome in separate data sources
HES: Hospital Episode Statistics for England; NDSCR: National Down Syndrome Cytogenetic Register. The denominator in HES is the estimated number of births in the birth cohort or number of HESIDs in the whole of HES, for each year of birth; the denominator for NDSCR is the estimated number of live births in England reported by the Office for National Statistics [1] (see *Estimation of prevalence and case ascertainment* for explanation)
Source: Hospital Episode Statistics (HES), NHS Digital (Copyright © 2019. Re-used with the permission of NHS Digital. All rights reserved) and the National Down Syndrome Cytogenetic Register (NDSCR), Public Health England.

Table S4 Geographic regions of linked and unlinked records

	Deterministic	Probabilistic (MW > 40.6)	Probabilistic (MW: 30.5–40.6)	Probabilistic (MW: 18.1–30.5)	Probabilistic (MW < 18.1)	Unlinked NDSCR records	Unlinked HES cases
n (NDSCR records)	4939	3694	449	662	534	137	–
n (HES records)	4941	3703	446	646	654	–	2280
NDSCR record[1]							
East Midlands	10.9%	9.0%	4.5%	4.5%	5.6%	10.0%	–
East of England	9.0%	10.0%	11.6%	7.0%	9.4%	8.3%	–
Greater London	20.8%	19.9%	27.2%	32.6%	28.9%	45.0%	–
North East	9.8%	9.0%	6.9%	10.3%	4.6%	< 8.0%	–
North West	14.6%	16.6%	17.8%	16.2%	19.6%	10.0%	–
South East	12.7%	12.4%	11.9%	13.6%	13.2%	< 8.0%	–
South West	10.3%	9.5%	7.4%	9.5%	7.6%	< 8.0%	–
West Midlands	11.9%	13.5%	12.6%	6.4%	11.0%	13.3%	–
HES record[1]							
East Midlands	10.8%	9.1%	4.1%	3.3%	8.0%	–	7.2%
East of England	8.9%	9.9%	10.5%	7.6%	6.0%	–	8.7%
Greater London	20.8%	20.0%	26.7%	27.5%	35.6%	–	26.6%
North East	10.0%	8.9%	6.8%	13.3%	5.3%	–	7.2%
North West	14.8%	16.7%	18.3%	25.4%	16.3%	–	19.6%
South East	12.6%	12.4%	12.3%	9.5%	12.3%	–	13.7%
South West	10.3%	9.5%	8.9%	8.3%	6.0%	–	7.2%
West Midlands	11.7%	13.5%	12.3%	5.1%	10.5%	–	9.8%

DOB: Date of birth; HES: Hospital Episode Statistics for England; MW: match weight; NDSCR: National Down Syndrome Cytogenetic Register.
NDSCR records exclude those with missing birth outcome. All data are column proportions, ignoring missing data, so that associations between region and linkage quality are reflected by differences in proportion across rows. Probabilistic links are grouped by 'match weight', a score reflecting the level of agreement over matching variables (see Methods). [1]The number of candidate links may be higher than the number of records in either file, indicating ambiguity of multiple links with equal agreement; for two of such candidate links, either at least one is false or both are true and it is the records in the contributing files that have not been completely deduplicated.
Source: Hospital Episode Statistics (HES), NHS Digital (Copyright © 2019. Re-used with the permission of NHS Digital. All rights reserved) and the National Down Syndrome Cytogenetic Register (NDSCR), Public Health England.

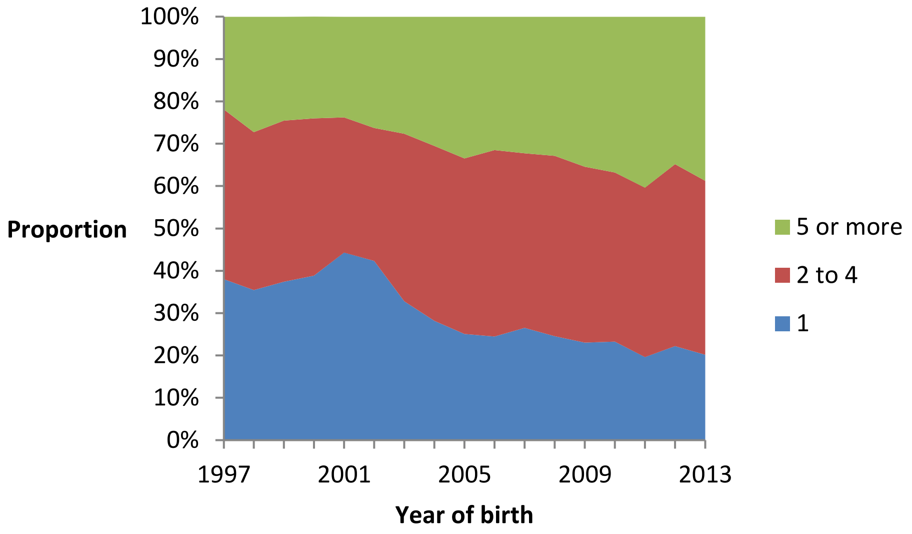

Figure S10 Proportion of HES cases, by number of episodes in first year of life, and year of birth

Source: Hospital Episode Statistics (HES), NHS Digital (Copyright © 2019. Re-used with the permission of NHS Digital. All rights reserved).

Appendix 3 References

1. Office for National Statistics. *Number of live births at home and total live births, England, 1994 to 2014 birth registrations*; 2016.
